# Development of a Tapping-Mode Scanning Probe Electrospray Ionization Platform for High-Sensitivity and Long-Term Stability in Single-Cell Mass Spectrometry Imaging of Tissue

**DOI:** 10.1021/acs.analchem.6c02386

**Published:** 2026-07-04

**Authors:** Takao Yasuda, Yoichi Otsuka, Tasuku Kato, Shuichi Shimma, Tomoki Misaka, Takuya Matsumoto, Michisato Toyoda

**Affiliations:** † Department of Physics, Graduate School of Science, 38419 The University of Osaka, 1-1 Machikaneyama-cho, Toyonaka, Osaka 560-0043, Japan; ‡ Department of Chemistry, Graduate School of Science, 320552 The University of Osaka, 1-1 Machikaneyama-cho, Toyonaka, Osaka 560-0043, Japan; § Forefront Research Center, Graduate School of Science, The University of Osaka, 1-1 Machikaneyama-cho, Toyonaka, Osaka 560-0043, Japan; ∥ Department of Biotechnology, Graduate School of Engineering, The University of Osaka, 2-1 Yamadaoka, Suita, Osaka 565-0871, Japan

## Abstract

To elucidate disease mechanisms, it is essential to develop analytical techniques capable of visualizing cellular heterogeneity in biological tissues. Mass spectrometry imaging (MSI) allows simultaneous visualization of multiple biomolecules in tissue sections in a single measurement. However, achieving single-cell (SC)-MSI requires high ion detection sensitivity and long-term ionization stability. Tapping-mode scanning probe electrospray ionization (t-SPESI) delivers a tiny amount of solvent from an oscillating fused silica capillary probe to the sample surface, enabling rapid extraction and ionization of analytes. In this study, we developed a t-SPESI measurement system and a chemical modification method for the probe surface to enable SC-MSI of biological tissues. Introducing a compact sample stage shortened the ion transfer tube to the mass spectrometer, thereby increasing ion detection sensitivity. Furthermore, forming a fluorine-containing molecular layer on the probe surface suppressed biomolecule adsorption at the probe tip and increased the long-term stability of solvent delivery. SC-MSI of mouse brain sections was performed at pixel sizes of 10 and 5 μm and visualized lipid distributions corresponding to fine tissue structures. These results demonstrate that the combination of system miniaturization and probe surface modification is an effective strategy for achieving high-sensitivity and long-term stable SC-MSI via t-SPESI, providing a robust platform for investigating cellular heterogeneity in biological tissues.

## Graphical abstract

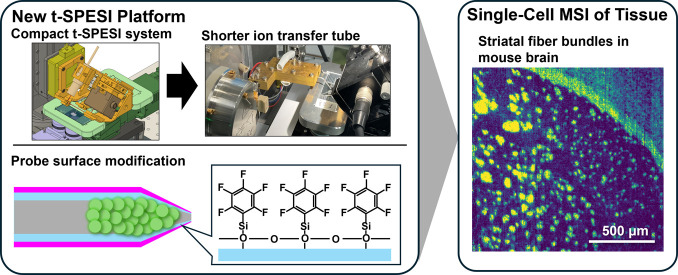

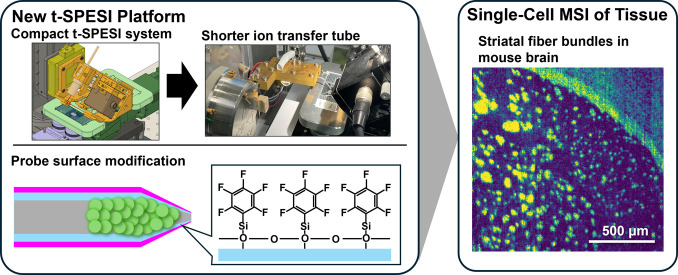

## Introduction

In biological tissues, functionally differentiated cells form highly organized hierarchical networks. Various intracellular and extracellular metabolic reactions are coordinated to maintain homeostasis. Cellular heterogeneity is closely associated with the onset and progression of various diseases, including neurodegenerative disorders,^1,2^ urological diseases,^3^ and aortic diseases.^4^ Therefore, to elucidate disease mechanisms and advance diagnostic and therapeutic methods, analytical techniques are required for visualizing cellular alterations in tissue sections at high spatial resolution.

Mass spectrometry imaging (MSI) enables label-free visualization of the spatial distribution of molecular components in biological samples. In MSI, analytes in microscopic regions of a tissue section are directly ionized and analyzed by mass spectrometer. By matching the spatial coordinates of the sample with the ion signal intensities, multiple ion images can be acquired in a single measurement. Single-cell (SC)-MSI, which visualizes molecular distributions in biological tissues at the cellular scale, has attracted considerable attention.^5–8^ The average diameter of mammalian cells ranges from 10 to 20 μm.^9^ Therefore, an ionization technique capable of probing microscopic regions of 10 μm or smaller is required to visualize cellular heterogeneity.

Secondary ion mass spectrometry (SIMS) uses a focused primary ion beam to irradiate the sample surface, inducing sputtering and generating secondary ions. A high-energy ion beam can achieve MSI with spatial resolutions on the order of several tens of nanometers; however, extensive fragmentation of biomolecules occurs, resulting in complex mass spectra.^10,11^ Cluster ion beams have been used to reduce fragmentation.^12^ A water cluster ion beam has been used for MSI of mouse cerebellum tissue at a pixel size of 6 μm and MSI of HeLa cells at a pixel size of 1 μm.^13^

Matrix-assisted laser desorption/ionization (MALDI) is widely used to ionize various biomolecules, including lipids, proteins, and nucleic acids.^14–16^ In MALDI, a matrix-coated sample is irradiated with a UV pulsed laser, and the matrix absorbs photon energy and undergoes electronic excitation, followed by rapid energy relaxation, local heating, ablation, and plume expansion, which desorbs matrix and analyte molecules. The analyte is then ionized in the expanding plume through proton-transfer reactions involving matrix-derived ions. MSI of mouse brain tissue at a pixel size of 1.4 μm^17^ and zebrafish embryos at a pixel size of 5 μm^18^ have been reported. To reduce the pixel size further, a transmission-mode configuration, in which the pulsed laser is irradiated from the backside of the sample, has been developed.^19^ Combining this configuration with post-ionization techniques increased the ion detection sensitivity, and MSI was performed on mouse cerebellum at a pixel size of 800 nm^20^ and mouse cerebellar cortex at a pixel size of 500 nm.^21^ In MALDI, the uniformity and thickness of the matrix crystals strongly affect spatial resolution; therefore, careful optimization is required.^22^

Ambient mass spectrometry using electrospray ionization (ESI) is characterized by minimal sample preparation and direct analysis. In desorption electrospray ionization (DESI), an electrospray focused by high-pressure nitrogen gas is directed onto the sample surface to desorb and ionize analytes. The lateral spread of the spray on the sample surface was suppressed by reducing the solvent flow rate to 250–500 nL/min, enabling MSI of rat brain and porcine spleen tissues at pixel sizes of 5–10 μm.^23^ Nanospray desorption electrospray ionization (nano-DESI) uses two capillaries in a V-shaped configuration to deliver solvent to the sample surface and perform ESI of the extracted analytes. In contrast to DESI, nano-DESI uses a shear-force probe and feedback control to maintain a constant distance between the capillary and sample surface. This approach decreases the effect of the sample topography on the extraction process and enables the simultaneous acquisition of the surface topography. Nano-DESI has been used for MSI of mouse brain tissue at a pixel size of 7 μm^24^ and MSI of mouse uterine tissue at a pixel size of 10 μm.^25^

We previously developed a tapping-mode scanning probe electrospray ionization (t-SPESI) system and applied the system to MSI.^26–29^ In this technique, a fused silica capillary probe oscillating vertically on the sample surface supplies a small amount of charged solvent to the surface. When the probe tip touches the sample, a liquid bridge is formed between them, enabling the extraction of analytes from the sample. Upon upward displacement of the probe, the extracted solution retained at the probe tip is subjected to ESI. In t-SPESI, the use of an oscillating probe enables the extraction and ionization of sample components with a substantially smaller amount of solvent than that used in DESI and nano-DESI. Therefore, t-SPESI can reduce the pixel size compared with DESI and nano-DESI. By monitoring the oscillation amplitude of the probe and using a feedback control system, fluctuations in the oscillation amplitude caused by sample surface irregularities are suppressed, enabling stable sampling and ionization.^27^ We have used t-SPESI to perform MSI of mouse testis tissue at a pixel size of 5 μm^29^ and of HeLa cells at a pixel size of 2 μm.^28^

As the pixel size in MSI decreases, the amount of analytes available for ionization decreases, and the total acquisition time increases.^30^ Therefore, to achieve SC-MSI of biological tissues using t-SPESI, techniques are required that improve ion detection sensitivity and long-term ionization stability. In a previously reported t-SPESI measurement system for SC-MSI of HeLa cells,^28^ the large sample stage of the inverted microscope and long ion transfer tube reduced the introduction efficiency of gas-phase ions generated by t-SPESI into the mass spectrometer. In addition, during prolonged measurements, the extracted materials accumulated at the probe tip, causing instability in the solvent flow.

In this study, we developed a t-SPESI measurement system equipped with a compact sample stage and a chemical modification method for the probe surface. We demonstrated that increasing ion detection sensitivity and suppressing the accumulation of materials at the probe tip improve SC-MSI of biological tissues.

## Materials and Methods

### Validation of the Effect of Ion Transfer Tube Length on Ion Signal Intensities

The ion transfer tube and heater block were fixed in front of the sampling cone of a quadrupole time-of-flight (TOF) mass spectrometer (Xevo G2-XS qTOF, Waters, USA). A stainless-steel tube (Kuroiwa Stainless Steel Industry Inc., Japan; outer diameter: 1.61 mm; inner diameter: 1.25 mm; material: SUS 304) was used as the ion transfer tube. The ion transfer tube was heated using a cartridge heater integrated into the heater block and a temperature controller (MTCS, Misumi, Japan). A NaI solution (400 mg/L in pure water/MeOH = 1:1, v/v) was delivered to a commercial silica emitter with a 10 μm tip orifice (FS360-20-10-N, New Objective, USA). The solution was infused using a syringe pump (Legato 185, KD Scientific, USA) at a flow rate of 300 nL/min. The silica emitter was connected to the syringe via a PEEK tube joined using a metal union. A voltage of 3.0 kV was applied to the metal union. The emitter tip position was adjusted to 1.2 mm from the end of the ion transfer tube while being monitored using a camera. Mass spectra were acquired for 30 s under each temperature condition. The peak intensities of the NaI cluster ions were calculated from the mass spectra averaged over the total acquisition time using MassLynx software (Waters).

### Polymer Beads for Fluorescence Imaging

Equal volumes of a 2.5% (w/v) suspension of monodisperse polystyrene latex beads (Fluoresbrite Polychromatic Red, ϕ6 μm; Fluoresbrite Yellow Green, ϕ10 μm, Polysciences, USA) were mixed, dropped onto a glass slide, and covered with a coverslip. Fluoresbrite Polychromatic Red beads were observed with a blue LED light source (HLV3-22BL-2C, CCS, Japan) combined with an excitation filter centered at 448 nm (VPFHT-25C-4480, Sigma Koki, Japan) and an emission filter centered at 526 nm (VPFHT-25C-5260, Sigma Koki). Fluoresbrite Yellow Green beads were observed with a green LED light source (HLV3-22GR-2C, CCS) with an excitation filter centered at 525 nm (VPFHT-25C-5250, Sigma Koki) and an emission filter centered at 588 nm (VPFHT-25C-5880, Sigma Koki).

### Capillary Probe Fabrication

Fused silica capillaries (TSP030375, Molex, USA; 360 μm outer diameter) were processed using a laser puller (P-2000, Sutter Instrument, USA) to fabricate capillary probes, as shown in Figure S1a–f. The operating parameters of the laser puller are listed in Table S1. To validate the feedback control system, a probe with a tip aperture diameter of 5 μm fabricated from a capillary with a 100 μm inner diameter was used. The capillary inner diameters and probe tip aperture diameters used for each MSI measurement are summarized in Table 1.

**Table 1:** Experimental Parameters for the MSI of Mouse Brain Sections

Data	MSI-1	MSI-2	MSI-3	MSI-4
Capillary inner diameter (μm)	100	30	100	30
Probe tip aperture diameter (μm)	7.5	1.3	8.4	3.2
Probe surface modification	None	None	PFPTES	PFPTES
X pitch (μm)	10.7	5.4	10.7	5.4
Y pitch (μm)	10.0	5.0	10.0	5.0
Measurement area (μm)	1600 × 2000	1400 × 615	5000 × 4500	1500 × 1500
Applied solvent voltage (kV)	3.1	2.0	6.5	4.0
Probe oscillation frequency (Hz)	502	548	570	549
Total measurement time (h)	3.3	1.8	17	7.2

### Chemical Modification of Fused Silica Probe and Substrate

A probe with a total length of 75 mm was treated with a UV–ozone cleaner (UV253MINI, Filgen, Japan) for 30 min. Triethoxy­(pentafluorophenyl)­silane (PFPTES; 10 μL, Tokyo Chemical Industry, Japan) was placed in a vial (186005666CV, Waters). The vial and the probe were mounted on a custom-made holder and placed in a 600 mL glass beaker (BR90648, DURAN, Germany) that was sealed with Parafilm. The bottom of the beaker was heated to 100 °C for 60 min on a hot plate to vaporize PFPTES.^31^ The beaker was allowed to cool to room temperature, and the probe was removed. The probe was mounted on a custom-made holder and heated at 100 °C for 30 min using a hot plate. The components and procedures used are shown in Figure S1g–l. For the contact angle measurements, synthetic fused silica substrates (10 × 10 × 0.5 mm, Labo-USQ, DAICO MFG, Japan) were ultrasonically cleaned in ultrapure water (Fujifilm Wako Pure Chemical Corporation, Japan), methanol (Nacalai Tesque, Japan), and ultrapure water for 15 min each, followed by air-drying for 30 min. The same procedure as for the probe was used to prepare substrates subjected to UV–ozone treatment and substrates chemically modified with PFPTES after UV–ozone treatment.

### Packing Silica Beads Inside the Probe

Amine-functionalized silica beads with average particle diameters of 10 μm (ReproSil-Pur 1000 NH_2_, Dr. Maisch, Germany) and 3 μm (SunArmor NH_2_, ChromaNik Technologies, Japan) were used. The 10 and 3 μm beads were packed into probes with tip aperture diameter of 8 and 2 μm, respectively (Figure S1m–o). Silica beads (200 mg) and methanol (1 mL) were mixed in a vial (186005666CV, Waters). The vial containing the suspension was placed in a packing device (NTMS-CP, Nikkyo Technos, Japan). The probe was cut about 5 mm from the rear end and fixed so that a 10 mm portion of the cut end was immersed in the suspension. The suspension was stirred using a magnetic stirrer, and nitrogen gas was applied at 6 MPa to pack the silica beads into the probe.

### XPS Analysis

X-ray photoelectron spectroscopy (XPS) measurements were performed on synthetic fused silica substrates subjected only to UV–ozone treatment and on synthetic fused silica substrates modified with PFPTES after UV–ozone treatment (ESCA 3057, ULVAC-PHI, Japan). XPS imaging of the probes treated using the same procedure as the synthetic fused silica substrates was performed (Kratos-ULTRA2, Shimadzu, Japan). In both XPS measurements, a monochromated Al Kα X-ray source and a charge neutralizer to suppress surface charging were used. A two-dimensional detector visualized the spatial distribution of the F 1s and C 1s signal intensities. The XPS spectra were analyzed by applying background correction using the iterative Shirley method, followed by peak fitting using a Voigt function (OriginPro 2026, OriginLab, USA).^32^

### Surface Free Energy Analysis

The static contact angles of solvents on the synthetic fused silica substrates subjected only to UV–ozone treatment and synthetic fused silica substrates modified with PFPTES after UV–ozone treatment were measured using a contact angle goniometer (CA-X, Kyowa Interface Science, Japan). Ultrapure water, diiodomethane, and *n*-hexadecane (1 μL each) were dropped onto the sample surface, and the contact angles were measured immediately after droplet deposition. The surface free energy was calculated using the Owens–Wendt method^33^ from the contact angles measured five times independently.

### TOF-SIMS Analysis

MSI of probes subjected only to UV–ozone treatment and probes modified with PFPTES after UV–ozone treatment was performed using a TOF-SIMS (TOF.SIMS5, IONTOF, Germany). An area of 150 × 150 μm was analyzed with a pixel size of 0.59 μm, followed by 2 × 2 pixel binning. A 60 keV Bi_3_^2+^ cluster ion beam was used as the primary ion source, and negative secondary ions were detected. Low-energy electrons were used to suppress surface charging of the samples.

### Preparation of Mouse Brain Sections

Fresh-frozen mouse brain sections (8 μm thick) were prepared using a cryomicrotome (CM1950, Leica, Germany) and mounted onto glass slides (MAS-01, Matsunami Glass, Japan). The tissue sections were stored at −80 °C in 50 mL Falcon centrifuge tubes (Corning, USA) containing silica gel. Before measurement, the tubes were allowed to return to room temperature, and the tissue sections were used without further treatment.

### MSI of Mouse Brain Sections

The extraction solvent was prepared by adding 0.1% formic acid to an equivalent volume mixture of *N*,*N*-dimethylformamide (LC grade, Nacalai Tesque) and methanol (LC grade, Nacalai Tesque). The solvent was delivered at a flow rate of 10 nL/min using a nanoflow pump (LC-20AD nano, Shimadzu). A fused silica capillary (TSP010375, Molex; length: 10 m; inner diameter: 10 μm; outer diameter: 360 μm) was connected between the nanoflow pump and the capillary probe to maintain a back pressure of approximately 5.9 MPa. The fused silica capillary was maintained at 40 °C in a column oven (CTO-20A, Shimadzu).

Measurements were performed in positive ion mode with an accumulation time of 200 ms, and mass spectra were acquired over an *m*/*z* range of 100–1200. For MSI at pixel sizes of 10 and 5 μm, probe scanning speeds were set to 50 and 25 μm/s, respectively. During the mass spectrometric acquisition, there was an interscan delay of 14 ms for each scan duration of 200 ms, resulting in an asymmetry between the pixel size in the probe scanning direction (X pitch) and that between the probe scanning lines (Y pitch). The measurement area, pixel size, applied solvent voltage, and probe oscillation frequency are listed in Table 1. The oscillation amplitude of the probe during scanning of the sample surface was set to 95% of its free oscillation amplitude. The temperatures of the heater block of the ion transfer tube and the orifice of the mass spectrometer were set to 150 °C. The mass resolving power of the mass spectrometer was >30,000 (full width at half-maximum; FWHM) in the sensitivity mode.

### Data Conversion and Analysis

Multiple raw-format data files acquired for each probe scan were integrated into a single raw-format file using a Perl script (Waters). The integrated raw data were subjected to lock-mass correction using HDI Imaging software (ver. 1.8, Waters), with PC 34:1 [M + H]^+^ at *m*/*z* 760.5851, an abundant lipid in mouse brain. The corrected raw data were then converted to imzML format, and the files were subsequently converted to IMDX format using IMDX Converter (Shimadzu). Ion images were generated using IMAGEREVEAL (Version 1.31.0.12906, Shimadzu). As a preprocessing step, signal intensities were normalized to the total ion current of each pixel. The resulting ion peak list was searched against the LIPID MAPS database^34^ to obtain level 2 putative lipid annotations.^35^ Protonated molecules ([M + H]^+^), sodium adduct molecules ([M + Na]^+^), and potassium adduct molecules ([M + K]^+^) were considered with a mass tolerance of *m*/*z* ± 0.005. Particle analysis was performed using ImageJ 1.54g (National Institutes of Health, USA). Because MS/MS analysis was not performed in this study, lipid annotations should be regarded as tentative annotations. Ion images preprocessed with a median filter were analyzed using the Analyze Particles function to calculate particle areas.^36^

## Results and Discussion

### Development of the Compact t-SPESI Measurement System

A three-dimensional rendering of the developed t-SPESI measurement system is shown in Figure 1a. The system comprises a t-SPESI unit, a sample stage unit, and a microscope unit mounted on a vibration isolation table. The isolation table is mounted on an aluminum frame rack, which houses the control unit. The control unit includes controllers for operating multiple automated stages for MSI, data input/output devices, and a personal computer for executing the control programs. The rack is equipped with wheels to allow coarse alignment between the mass spectrometer and the t-SPESI system. The instruments used in this system are listed in Table S2.

**Figure 1:**
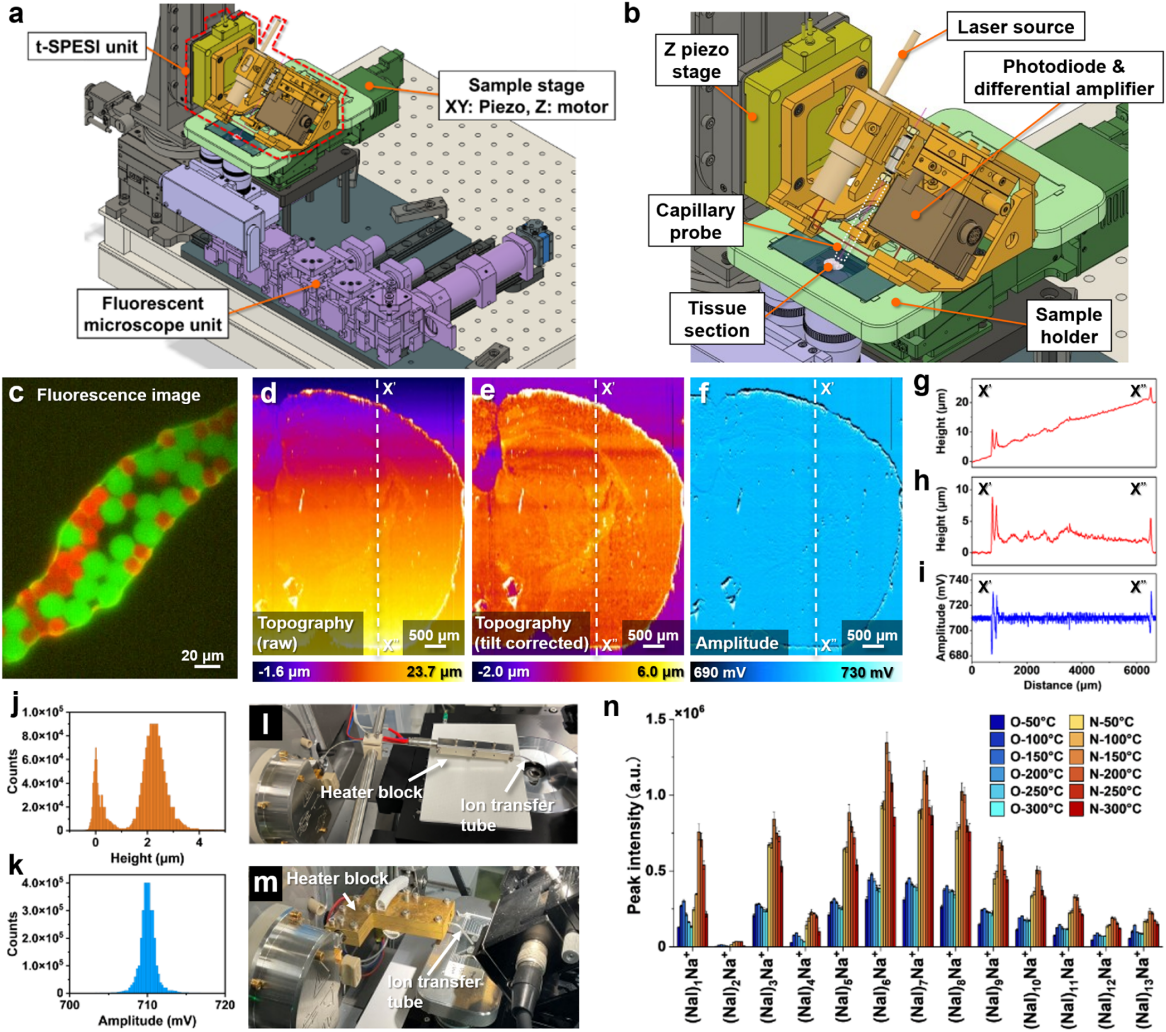
(a) Rendered image of the developed measurement system. (b) Enlarged view of the t-SPESI unit and the sample stage unit. (c) Fluorescence microscope images of two types of fluorescent beads. (d) Topographic image of a mouse brain section (uncorrected). (e) Topographic image of the mouse brain section after tilt correction. (f) Amplitude image of the mouse brain section. (g–i) Section profiles along the dashed lines shown in (d–f). (j) Histogram of the sample height after tilt correction. (k) Histogram of the probe oscillation amplitude. (l) Photograph of the conventional ion transfer tube. (m) Photograph of the developed ion transfer tube. (n) Comparison of the signal intensities of NaI cluster ions. In the legend, O and N indicate the results obtained using the ion transfer tubes shown in (l) and (m), respectively, and the numbers indicate the heater temperature.

An enlarged view of the t-SPESI and sample stage units is shown in Figure 1b. The t-SPESI unit includes a laser light source, a piezoelectric actuator for probe excitation, and a photodiode unit all fixed in a single housing. The t-SPESI unit is mounted on the Z-piezo stage. Compared with the previous configuration,^28^ the frame, XY stage for photodiode alignment, and laser source are miniaturized, resulting in a 45% reduction in the total weight (from 1065 to 583 g). The weight reduction is expected to increase the responsiveness of the feedback control system.

The sample stage unit comprises an ultrasonic piezo motor-driven XY stage (travel range: 22 mm; minimum step size: 0.2 μm; bidirectional repeatability: ± 0.2 μm), a stepper motor-driven Z stage (travel range: 5 mm; minimum step size: 0.1 μm; repeatability: 1 μm), and a custom-designed sample holder (green region in Figure 1a). The aluminum sample holder contains a groove for fixing a glass slide. Compared with a previously used commercial motorized microscope stage,^27^ the footprint of the stage was greatly reduced. Consequently, the horizontal distance from the optical focal point to the edge of the stage was reduced by 80% (from 160 to 31.5 mm).

The microscope unit is a compact inverted fluorescence microscope (purple region in Figure 1a). The two objective lenses can be interchanged. The system can acquire bright-field images with top illumination and fluorescence images with two excitation wavelengths. An example of fluorescence imaging using fluorescent beads is shown in Figure 1c. Fluorescence imaging is expected to be useful for future SC-MSI. In this study, bright-field imaging was used to align the probe tip with the sample.

Tissue sections exhibit microscopic surface roughness that can tilt the sample stage slightly. When a probe scans the sample surface, the oscillation amplitude depends on surface height. Variations in oscillation amplitude alter the formation time of the liquid bridge and electrospray, thereby reducing measurement stability and reproducibility. Therefore, a feedback control mechanism to maintain a constant oscillation amplitude is essential.^27^ The system incorporates a feedback control mechanism similar to the previously reported mechanism.^27^ A laser beam is directed onto the side of the probe, and the displacement of the probe shadow is detected using a photodiode unit. The displacement signal is processed using a lock-in amplifier to measure the amplitude. A proportional–integral control signal corresponding to the deviation from the set point amplitude is used to adjust the vertical position of the t-SPESI unit continuously.

To validate the feedback control mechanism, mouse brain sections were measured using a dry probe without solvent delivery. The uncorrected topography image (Figure 1d) showed different color tones between the upper and lower regions, indicating an overall sample tilt. After tilt correction of the glass substrate, the topographic image (Figure 1e) showed uniform contrast in the glass region, and structures approximately 1 μm higher than those in the surrounding regions were observed in the corpus callosum, fimbria, and internal capsule. The amplitude image (Figure 1f) showed uniform contrast in the tissue region. The section profile of Figure 1d (Figure 1g) revealed that the overall tilt angle of the sample was 0.17°. The section profile of Figure 1e (Figure 1h) confirmed that the mouse brain section was higher than the glass substrate. The section profile of Figure 1f (Figure 1i) showed amplitude fluctuations near the boundary between the glass substrate and the tissue owing to the response delay in the feedback control system. However, during probe scanning on the tissue section, the amplitude variation was suppressed to 14 mV. Histogram analysis was conducted to evaluate the height and amplitude variations across the entire image. In the histogram of the corrected sample height (Figure 1j), distinct peaks corresponding to the glass substrate and brain section heights were observed. In the oscillation amplitude histogram (Figure 1k), a peak centered at the amplitude set point (710 mV) was observed, with a FWHM of 1.3 mV, corresponding to 0.18% of the oscillation amplitude. These results demonstrated that the feedback control system worked properly.

The distance between the mass spectrometer and the t-SPESI unit was reduced by miniaturizing the sample stage. Consequently, the ion transfer tube length was reduced by 56% compared with the conventional ion transfer tube^28^ (from 270 mm in Figure 1l to 120 mm in Figure 1m). To evaluate the effects of the ion transfer tube length and temperature on ion signal intensity, NaI cluster ions generated by ESI were analyzed at temperatures ranging from 50 to 300 °C for each ion transfer tube (Figure 1n). The mass spectra obtained under all conditions are shown in Figure S2. Shortening the ion transfer tube increased the NaI cluster ion intensity by an average of 2.5-fold (range: 1.5–5.3-fold). This increase was attributed to the decreased residence time of the charged droplets and ions in the tube, leading to fewer wall collisions and lower ion loss.^37^ The cluster ion intensity reached a maximum at 150 °C. Increasing the temperature from 50 °C increased ion intensity by accelerating the desolvation of charged droplets and increasing gas-phase ion production. Above 150 °C, the signal intensity decreased, probably because of thermal choking and a transition to turbulent flow,^38^ which decreased the gas velocity, increased the residence time, and increased droplet and ion loss through wall collisions. The temperature yielding the maximum intensity (150 °C) was lower than previously reported^28^ (250 °C), which may be caused by differences in the NaI solution concentration (400 mg/L vs 2 g/L) and emitter tip diameter (10 vs 25 μm). The lower molecular concentration and smaller droplet volume of the initially generated charged droplets probably decreased the thermal energy required for desolvation. Similar trends were also observed in negative ion mode, where shortening the ion transfer tube increased the NaI cluster ion signal intensity by an average of 2.9-fold (1.7–4.8-fold), and the signal intensity reached a maximum at 150 °C (Figure S3).

### Instability of Ionization in MSI of Tissue Using Untreated Capillary Probes

We performed SC-MSI of mouse brain sections using the developed system. SC-MSI was conducted with pixel sizes of 10 and 5 μm. To form a liquid bridge smaller than the pixel size and thereby achieve localized extraction in each pixel, a probe with a tip aperture diameter smaller than the pixel size must be used. First, the results of MSI at a pixel size of 10 μm in the hippocampal region of the mouse brain are discussed, and the measurement area is shown in Figure 2a. A probe with a tip aperture diameter of 7.5 μm, fabricated from a capillary with an inner diameter of 100 μm, was used. The probe was fabricated using a laser puller and packed with silica beads. In the ion image at *m*/*z* 798.542 (Figure 2b), the ion signal intensity fluctuated over time during the 3.3 h measurement period. This ion was putatively assigned to phosphatidylcholine 34:1 [M + K]^+^. Optical microscope images of the region near the probe tip before and after MSI were compared. The probe before measurement (Figure 2c) exhibited a sharp tip, whereas after measurement, deposits at the tip and accumulation of materials inside the probe were observed (Figure 2d). Therefore, components extracted by the solvent and the debris released from the biological tissue accumulated at the probe tip, causing fluctuations in the solvent flow and resulting in temporal variations in the ion signal intensity in the ion images.

**Figure 2:**
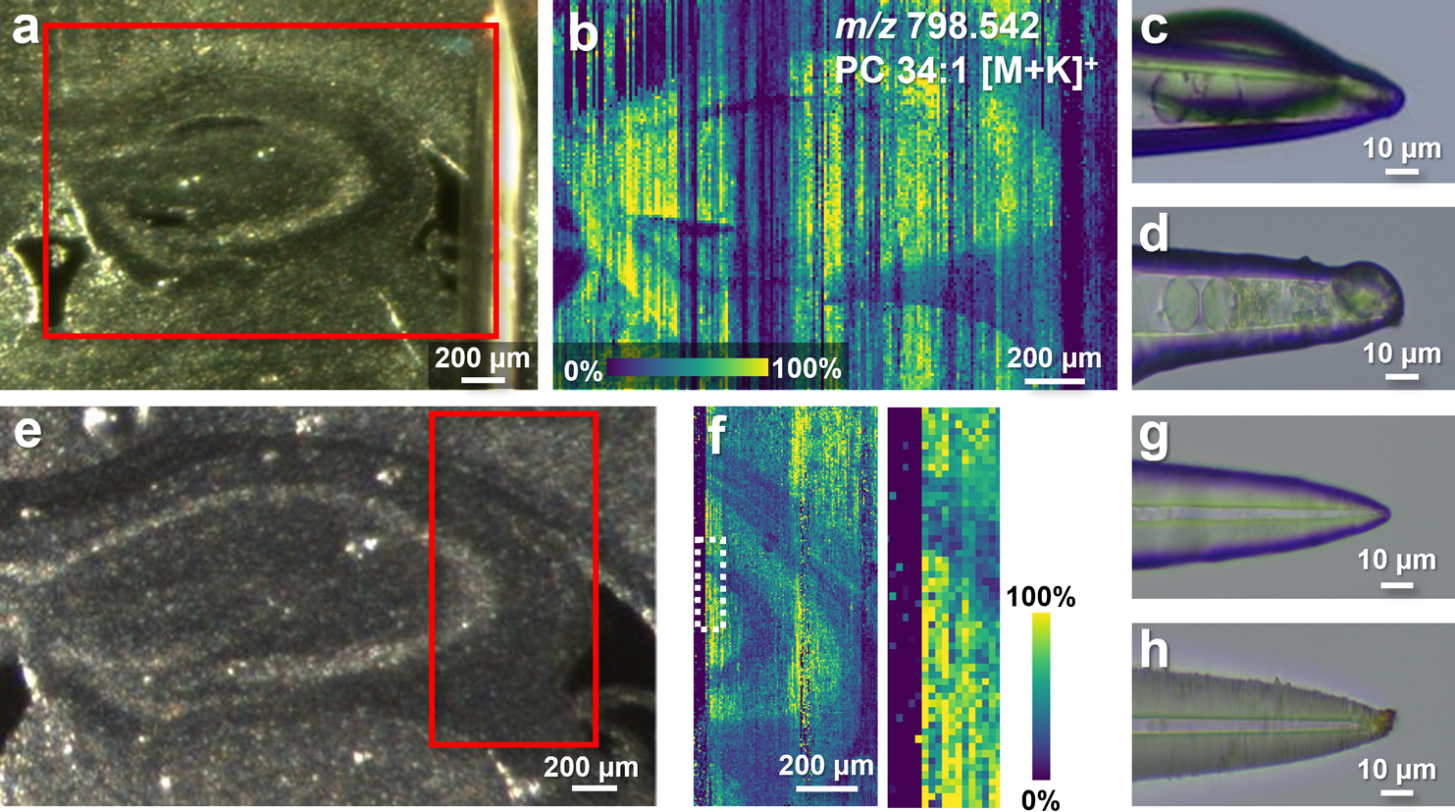
(a) Optical microscope image of a mouse brain section. The red box indicates the region where MSI was performed. (b) Ion image at *m*/*z* 798.542 acquired at a pixel size of 10 μm. (c, d) Optical microscope images of the probe tip before (c) and after (d) MSI. (e) Optical microscope image of another mouse brain section. The red box indicates the region where MSI was performed. (f) Ion image at *m*/*z* 798.542 acquired at a pixel size of 5 μm. An enlarged view of the dashed region is shown on the right. (g, h) Optical microscope images of the probe tip before (g) and after (h) MSI.

Next, the results of MSI at a pixel size of 5 μm in the hippocampal region of another mouse brain section (Figure 2e) are discussed. A probe with a tip aperture diameter of 1.3 μm, fabricated from a capillary with an inner diameter of 30 μm, was used. In the ion image at *m*/*z* 798.542 (Figure 2f), the ion signal intensity fluctuated during each probe scan and disappeared after 124 scan lines. Comparison of the optical microscope images of the probe tip before and after measurement (Figure 2g, h) revealed deposits at the probe tip after measurement. The deposits caused complete blockage of the probe aperture, stopping the solvent flow. These results suggest that, although reducing the probe tip aperture diameter is necessary for pixel-size miniaturization in SC-MSI, a smaller aperture is more susceptible to clogging by extracted components and tissue debris, which is a critical problem for stable measurement.

The biological components adhering to the tip of the fused silica probe used for t-SPESI probably included proteins and lipids. In the adsorption of proteins onto fused silica surfaces, single-molecule adsorption occurs in the initial stage, followed by cluster formation nucleated by the adsorbed molecules.^39^ In the initial stage, hydrogen bonding between the carbonyl, imide, and amino groups of proteins and the silanol groups on the fused silica surface is dominant.^39^ For lipid molecules, hydrogen bonding between phosphate and choline groups is the dominant interaction.^40,41^

To achieve SC-MSI, the probe tip aperture diameter and solvent flow rate must be decreased to minimize the spread of the liquid bridge; therefore, we investigated strategies to prevent the adsorption of biological components on probes and achieve long-term ionization stability.

### Fabrication of a Fluorine-Containing Molecular Layer on the Probe Surface

To suppress the adhesion of biomolecules to the probe surface, which interferes with the long-term stability of the extraction–ionization process, we formed a molecular layer of PFPTES (Figure 3a) on the probe surface. PFPTES modification has been reported for silicon probes used in atomic force microscopy^31^ and for titanium needles used in probe ESI.^42^ In both approaches, condensation reactions between the hydroxyl groups on the solid surface and PFPTES were used to inactivate the solid surface and reduce its adsorption affinity for molecules. To form the molecular layer, organic contaminants on the fused silica surface were removed by UV–ozone treatment, PFPTES was adsorbed onto the surface by vapor deposition, and a dehydration–condensation reaction was induced by heating.

**Figure 3:**
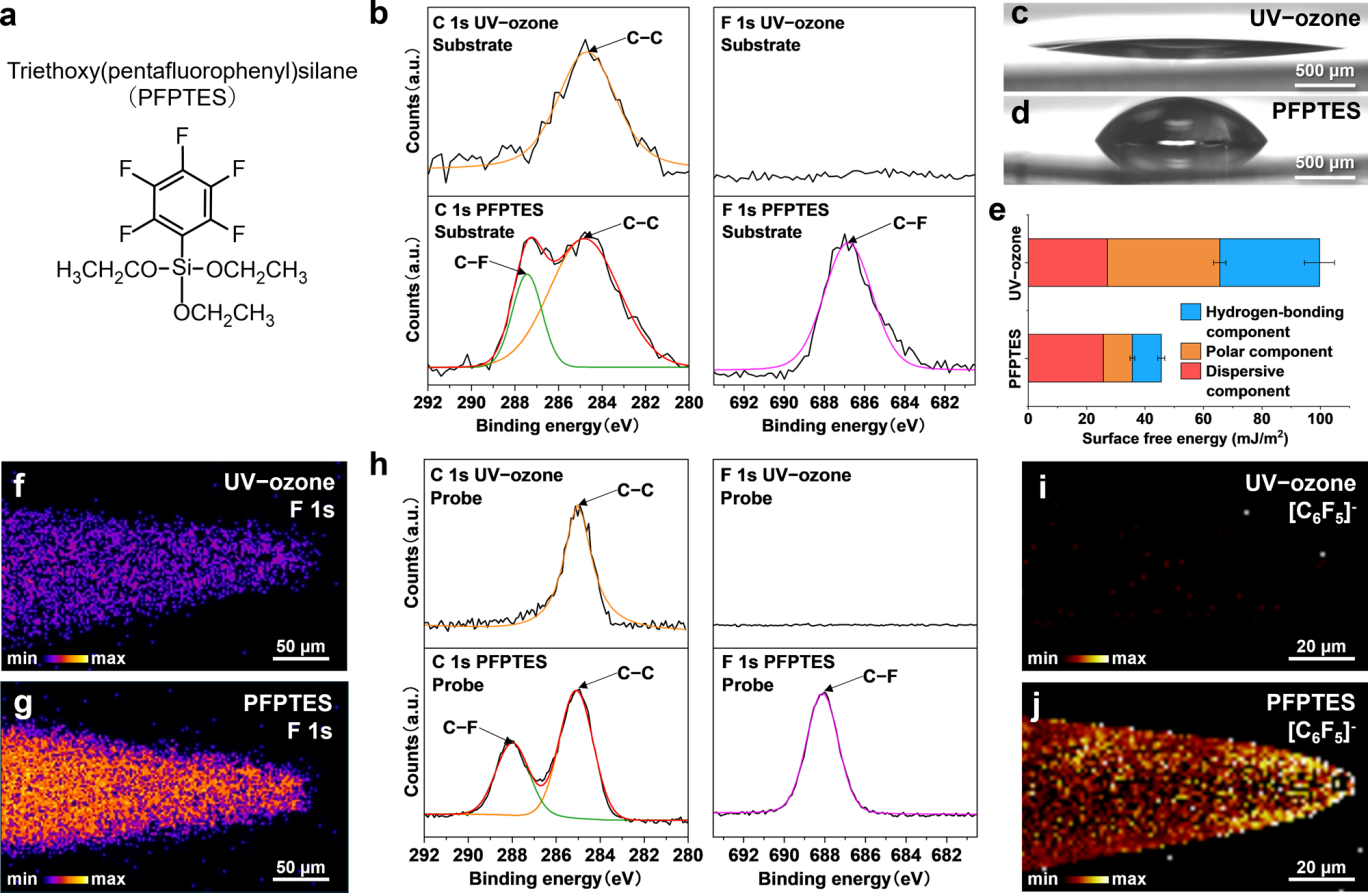
(a) Chemical structure of PFPTES. (b) XPS C 1s and F 1s spectra of UV–ozone-treated synthetic fused silica substrates and PFPTES-modified synthetic fused silica substrates. (c, d) Comparison of the static contact angles of ultrapure water on (c) UV–ozone-treated synthetic fused silica substrates and (d) PFPTES-modified synthetic fused silica substrates. (e) Comparison of surface free energy. (f, g) F 1s intensity images obtained by XPS imaging of (f) UV–ozone-treated probes and (g) PFPTES-modified probes. (h) XPS C 1s and F 1s spectra of UV–ozone-treated probes and PFPTES-modified probes. (i, j) TOF-SIMS ion images of [C_6_F_5_]^−^ obtained from (i) UV–ozone-treated probes and (j) PFPTES-modified probes.

To verify that PFPTES formed covalent bonds with the synthetic fused silica surface and modified its surface properties, a molecular layer was fabricated on synthetic fused silica substrates, followed by surface analysis using XPS. Figure 3b shows the C 1s and F 1s spectra of the synthetic fused silica substrates subjected to UV–ozone treatment and PFPTES modification. In the C 1s spectra, a peak at 284 eV was observed for the UV–ozone-treated and PFPTES-modified synthetic fused silica substrates. This peak was attributed to C–C bonds from organic contaminants adsorbed on the synthetic fused silica surface upon exposure to the atmosphere. In contrast, a peak at 288 eV was observed only for the PFPTES-modified substrate and corresponded to the binding energy of C–F bonds.^43^ In the F 1s spectra, no peak was detected for the UV–ozone-treated substrate, whereas a distinct peak at 687 eV, corresponding to C–F bonds, was observed for the PFPTES-modified substrate. These results suggest that PFPTES was bonded to the synthetic fused silica substrate surface.

The surface free energy was evaluated using contact angle measurements. The water contact angles on the UV–ozone-treated and PFPTES-modified synthetic fused silica substrates were 6.1° and 53.4°, respectively (Figure 3c, d), indicating that the PFPTES layer increased hydrophobicity. The contact angles for diiodomethane and *n*-hexadecane were also measured (Figure S4), and the surface free energy was calculated using the Owens–Wendt method^33^ (Figure 3e). Compared with the UV–ozone-treated substrate, the PFPTES-modified synthetic fused silica substrate exhibited a decrease of about 75% in the polar and hydrogen-bonding components of the surface free energy.

To verify the PFPTES modification of the probe surface used in t-SPESI, XPS imaging was performed. In the UV–ozone-treated probe, a weak F 1s signal was observed along the probe geometry (Figure 3f). In XPS imaging of three-dimensional structures, local surface tilting can affect the photoelectron emission angle and collection efficiency, resulting in a geometry-dependent contrast.^44^ Therefore, the apparent probe-shaped contrast observed in Figure 3f is attributed to variations in the background signal intensity influenced by the probe geometry. In contrast, the PFPTES-modified probe exhibited a clear and strong F 1s signal distribution corresponding to the probe geometry (Figure 3g), indicating a uniform presence of fluorine-containing molecules on the probe surface. Figure 3h shows the C 1s and F 1s spectra of the UV–ozone-treated and PFPTES-modified probes. In the C 1s spectrum of the PFPTES-modified probe, peaks were observed at 286.7–287.5 eV, consistent with those detected on the synthetic fused silica substrate. In addition, the F 1s spectrum showed a peak around 688 eV, similar to that observed for the PFPTES-modified synthetic fused silica substrate. Furthermore, TOF-SIMS was used for MSI. In the UV–ozone-treated probe, the fragment ion [C_6_F_5_]^−^ was not observed (Figure 3i), whereas this ion was detected in the PFPTES-modified probe (Figure 3j). These results indicate that PFPTES was bonded to the probe surface.

### Increased Stability of SC-MSI of Tissue Using Chemically Modified Probes

To evaluate the effectiveness of PFPTES-modified probes in MSI of biological tissues, SC-MSI was performed on mouse brain sections centered on the caudoputamen region using two probes with different tip apertures. The MSI results acquired at a pixel size of 10 μm are shown in Figure 4a–d. Figure 4a shows an optical microscope image of the section, and MSI was performed in the region indicated by the box. The total measurement time was 17 h. The ion image of *m*/*z* 838.617 is shown in Figure 4b, and additional ion images are shown in Figure S5. This ion was putatively assigned to hexosylceramide (HexCer) 40:1; O3 [M + K]^+^. The other ion peaks were assigned as shown in Table S3. HexCer is a lipid that is important in stabilizing myelin structure and regulating glial–axon signaling,^45^ and HexCer is involved in neurodegenerative diseases, such as Alzheimer’s disease^46^ and Parkinson’s disease.^47^ In the ion images, localized signal intensities were observed in the anterior commissure, corpus callosum, and parts of the striatum. No large temporal fluctuations in ion signal intensity were observed during long-term measurements. Optical microscope images of the probe tip before and after MSI are shown in Figure 4c, d. No noticeable changes were observed at either probe tip, indicating that PFPTES on the probe surface suppressed the adhesion of extracted components and debris. Figure 4e shows an enlarged ion image of the selected region in Figure 4b. Myelinated fiber bundles in the striatum are formed by clusters of myelinated corticofugal fibers that descend through densely packed gray matter. In coronal sections, these fiber bundles appear as island-like structures with sizes from several to several tens of micrometers.^48^ The localized distribution of HexCer likely reflects the island-like fiber bundle structures.

**Figure 4:**
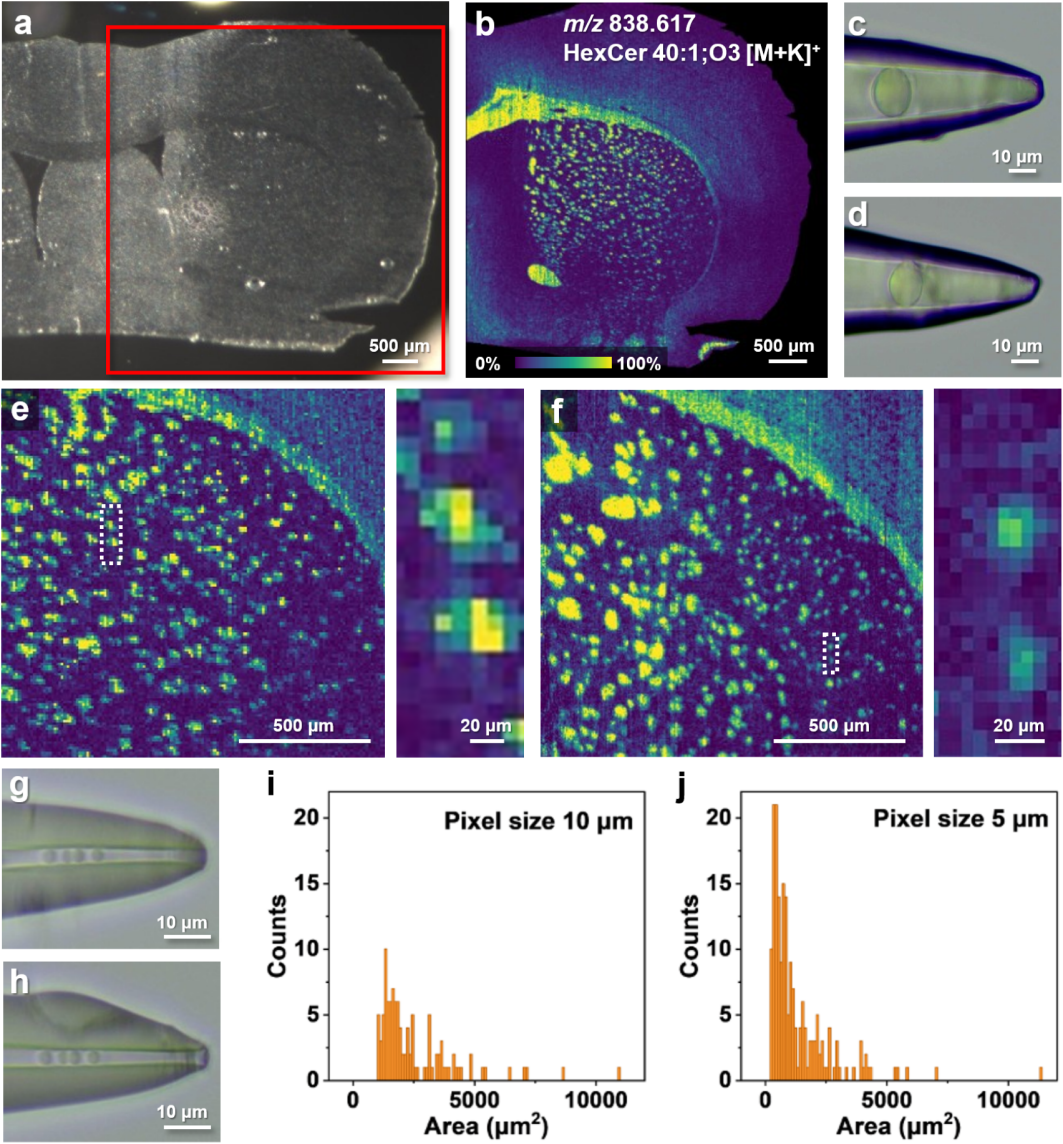
(a) Optical microscope image of a mouse brain section. MSI was performed in the region indicated by the red box. (b) Ion image of *m*/*z* 838.617 acquired at a pixel size of 10 μm. (c, d) Optical microscope images of the probe tip (c) before and (d) after MSI, corresponding to (b). (e) Enlarged ion image of the striatal region shown in (b). (f) Ion image of *m*/*z* 838.617 acquired at a pixel size of 5 μm. (g, h) Optical microscope images of the probe tip (g) before and (h) after MSI, corresponding to (f). (i, j) Histogram of the areas of the island-like regions in (e) and (f), respectively.

Figure 4f shows the ion image of *m*/*z* 838.617 acquired using MSI with a pixel size of 5 μm. The total measurement time was 7 h. Compared with the ion image acquired at a pixel size of 10 μm, the island-like fiber bundle structures were more clearly resolved at a pixel size of 5 μm. Optical microscope images of the probe before and after MSI are shown in Figure 4g, h. No major deposits were observed at the probe tip after the measurements.

Particle analysis was performed on the ion images shown in Figure 4e and f. In the ion image acquired at a pixel size of 10 μm (Figure 4e), 108 island-like structures were detected, with a minimum area of 1009.2 μm^2^ (Figure 4i). In contrast, in the ion image acquired at a pixel size of 5 μm (Figure 4f), 173 island-like structures were detected, with a minimum area of 240.2 μm^2^ (Figure 4j). These results indicate that reducing the pixel size enabled the separation and visualization of closely spaced fine fiber bundle structures.

The t-SPESI platform developed in this study enabled SC-MSI of mouse brain tissue at a pixel size of 5 μm, which is comparable to the pixel sizes reported for the MSI of biological tissues using low solvent flow DESI^23^ and nano-DESI.^25^ The improved ion detection sensitivity and long-term stability using a chemically modified probe in the present platform with feedback control suggest that, with further optimization of the probe tip aperture diameter, solvent flow rate, and liquid bridge size, t-SPESI has the potential to achieve MSI of biological tissues at a pixel size on the order of 2 μm or less.

The suppression of biomolecular adsorption on the PFPTES-modified probe surface suggests that the interaction between PFPTES and biomolecules is weak. On fused silica surfaces modified with fluorinated compounds, the fluorous effect promotes preferential F–F interactions, greatly decreasing interactions with biomolecules.^49^ The termination of a fused silica surface with pentafluorophenyl groups suppresses hydrogen bonding between proteins or lipid molecules and the substrate surface, inhibiting irreversible adsorption.^50^ Furthermore, probe oscillation and continuous solvent flow during measurement probably desorbed weakly adsorbed biomolecules, preventing the accumulation of biological components at the probe tip, even during long-term measurements.

In t-SPESI, interactions between the extraction solvent forming the liquid bridge and the probe or sample surface can affect liquid bridge formation and the size of the initially charged droplets generated during ESI. Therefore, understanding and controlling the physicochemical properties of tiny amounts of solvent at the solid–liquid interface is critical for achieving high-spatial-resolution and high-sensitivity SC-MSI. To the best of our knowledge, this study is the first report of MSI performed using a capillary probe modified with a molecular layer. Silane coupling reactions are a versatile surface modification method that enables the covalent attachment to fused silica surfaces of a wide variety of molecules with diverse chemical structures and physicochemical properties.^51^ A well-defined extraction–ionization process requires the application of surface science concepts, including molecular modification. To achieve further long-term stabilization and improve spatial resolution, it is important to investigate surface treatment methods, such as other silane coupling reagents, that can simultaneously suppress the adsorption of extracts and miniaturize the liquid bridge.

## Conclusions

We developed a compact t-SPESI measurement system and a chemical surface modification method for capillary probes to enable SC-MSI of biological tissues. By miniaturizing the sample stage compared with the previous configuration, the ion transfer tube length was shortened, resulting in increased ion detection sensitivity. In addition, a PFPTES molecular layer was formed on the probe surface. Using the developed measurement system and PFPTES-modified probes, proof-of-concept SC-MSI of mouse brain sections demonstrated stable measurements over extended periods, with suppressed temporal fluctuations in ion signal intensity caused by flow path blockage. These results suggest that chemical modification of the probe surface stabilizes direct extraction–ionization processes using a capillary probe and solvent. Integrating surface modification approaches developed in surface science into ambient pressure sampling ionization techniques is expected to contribute to improved data quality in SC-MSI of biological tissues.

## Supplementary Material



## Notes

Detailed protocols for capillary probe fabrication, chemical modification, and bead loading; mass spectra and signal intensity comparisons of NaI cluster ions; contact angle measurements; additional ion images of mouse brain sections; and instrumental parameters (PDF)
