# Supplementary material for: Development of a Tapping-Mode Scanning Probe Electrospray Ionization Platform for High-Sensitivity and Long-Term Stability in Single-Cell Mass Spectrometry Imaging of Tissue
Source: Anal Chem. 2026 Jul 4;98(27):20520–30. doi: 10.1021/acs.analchem.6c02386 (PMC13373919; doi:10.1021/acs.analchem.6c02386)
Supplement: Supplementary file 1 [file ac6c02386_si_001.pdf]

Supporting Information

Development of a Tapping-Mode Scanning Probe Electrospray Ionization Platform for High-Sensitivity and Long-Term Stability in Single-Cell Mass Spectrometry Imaging of Tissue

Takao Yasuda[1], Yoichi Otsuka[1,2,3†], Tasuku Kato[1], Shuichi Shimma[4], Tomoki Misaka[2], Takuya Matsumoto[2], and Michisato Toyoda[1,2,3]

[1] Department of Physics, Graduate School of Science, The University of Osaka, 1-1 Machikaneyama-cho, Toyonaka, Osaka 560-0043, Japan

[2]Department of Chemistry, Graduate School of Science, The University of Osaka, 1-1 Machikaneyama-cho, Toyonaka, Osaka 560-0043, Japan

[3]Forefront Research Center, Graduate School of Science, The University of Osaka, 1-1 Machikaneyama-cho, Toyonaka, Osaka 560-0043, Japan

[4]Department of Biotechnology, Graduate School of Engineering, The University of Osaka, 2-1 Yamadaoka, Suita, Osaka 565-0871, Japan

†Corresponding author

†Email: otsuka@phys.sci.osaka-u.ac.jp

Contents:

Figure S1. Protocol for fabrication, chemical modification, and bead loading of the capillary probe.

Figure S2. Comparison of the mass spectra of NaI cluster ions obtained using ion transfer tubes of different lengths.

Figure S3. Comparison of the signal intensities of NaI cluster ions obtained using ion transfer tubes of different lengths.

Figure S4. Comparison of contact angles of diiodomethane and *n*-hexadecane on quartz substrates.

Figure S5. Ion images of a mouse brain section.

Table S1. Parameters of the laser puller used to fabricate the capillary probe.

Table S2. List of instruments used in the t-SPESI measurement system.

Table S3. Putative assignments of positive ion peaks observed in the mouse brain tissue.

Figure S1. Protocol for fabrication, chemical modification, and bead loading of the capillary probe.

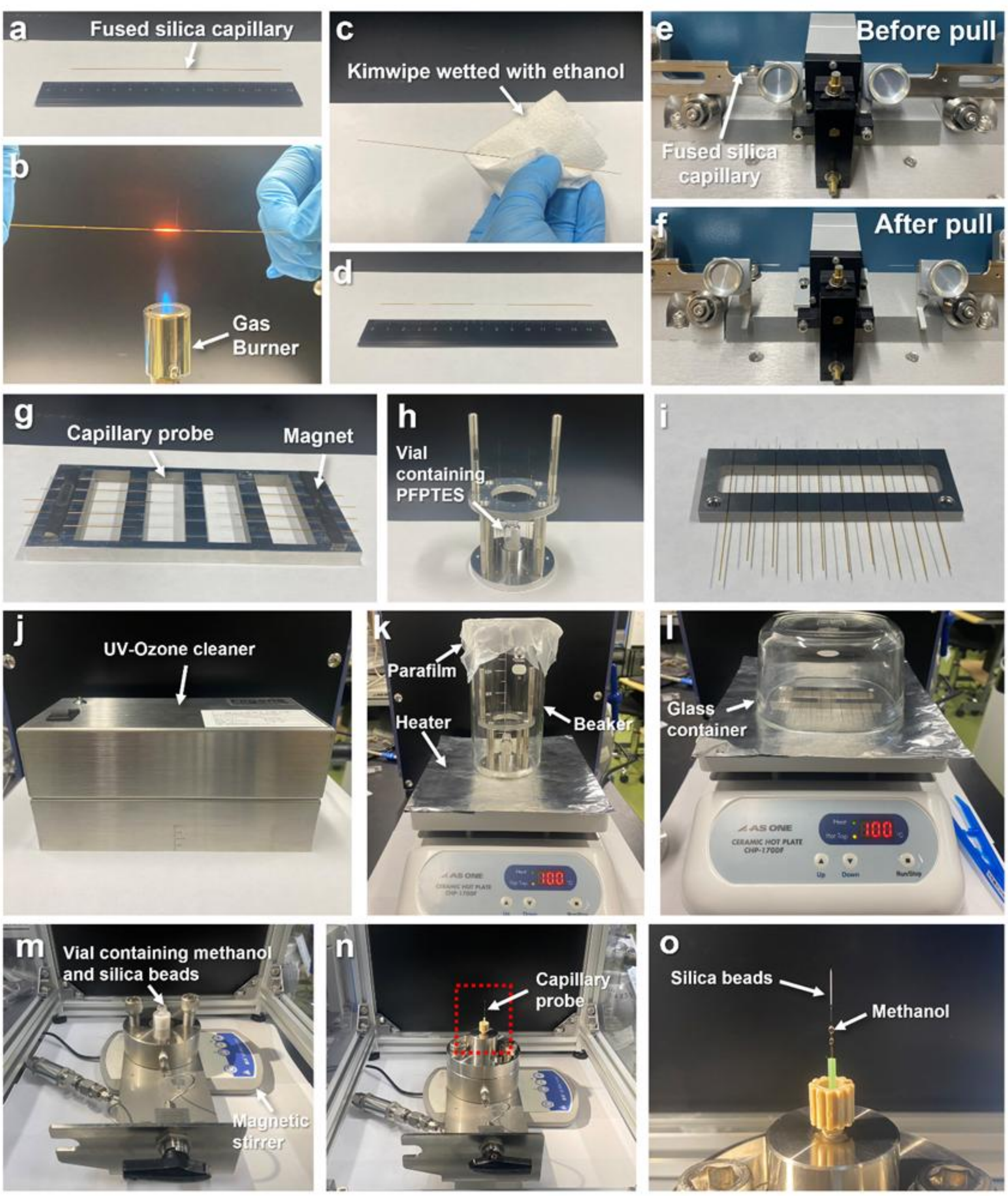

(a–f) Protocol for fabricating the capillary probe. (a) A fused silica capillary (TSP030375, Molex, USA; 360 µm outer diameter) is cut to a length of approximately 15 cm using a capillary cutter. (b) To remove the polyimide coating in the center of the capillary where the laser is applied, the coating approximately 1 cm on each side of the center is carbonized by heating with a gas burner. (c) The carbonized polyimide coating is removed using a Kimwipe soaked in ethanol. (d) The quartz surface at the center of the capillary is exposed. (e) The capillary is mounted on a laser puller (P-2000, Sutter

Instrument, USA). (f) After entering the laser puller parameters, laser irradiation and bidirectional pulling are performed to fabricate two capillary probes. (g) Probe holder for UV–ozone cleaning. (h) Probe holder for PFPTES (Tokyo Chemical Industry, Japan) vapor deposition. (i) Probe holder for heat treatment. (j) The probe is placed on the probe holder shown in (g) and is treated for 30 min using a UV-ozone cleaner (UV253MINI, Filgen, Japan). (k) The probe and a vial containing 10 μL of PFPTES are placed on the vapor deposition probe holder shown in (h) and are enclosed in a 600 mL glass beaker (BR90648, DURAN, Germany) sealed with Parafilm. The bottom of the beaker is heated at 100 °C for 60 min using a hot plate to vaporize PFPTES. After heating, the system is cooled at room temperature for 30 min. (l) The probe is placed on the heat treatment probe holder shown in (i) and is covered with a glass container. The bottom of heat treatment probe holder shown in (i) is heated at 100 °C for 30 min using a hot plate. The heating is then stopped, and the probe is allowed to cool for 60 min. (m–o) Protocol for bead packing of the probe. (m) Silica beads (20 mg, ReproSil-Pur 1000 NH_2, Dr. Maisch, Germany), methanol (1000 μL), and a stir bar are placed in a vial and are stirred for 20 min using the magnetic stirrer at the bottom of the bead-loading apparatus. (n) The probe is fixed in the bead-loading apparatus so that approximately 10 mm of the probe tip is immersed in the bead suspension and is pressurized with nitrogen gas at 6 MPa. (o) The applied pressure delivers the bead suspension to the probe tip, where silica beads accumulate. The solvent flowing from the probe tip is confirmed.

Figure S2. Comparison of the mass spectra of NaI cluster ions obtained using ion transfer tubes of different lengths.

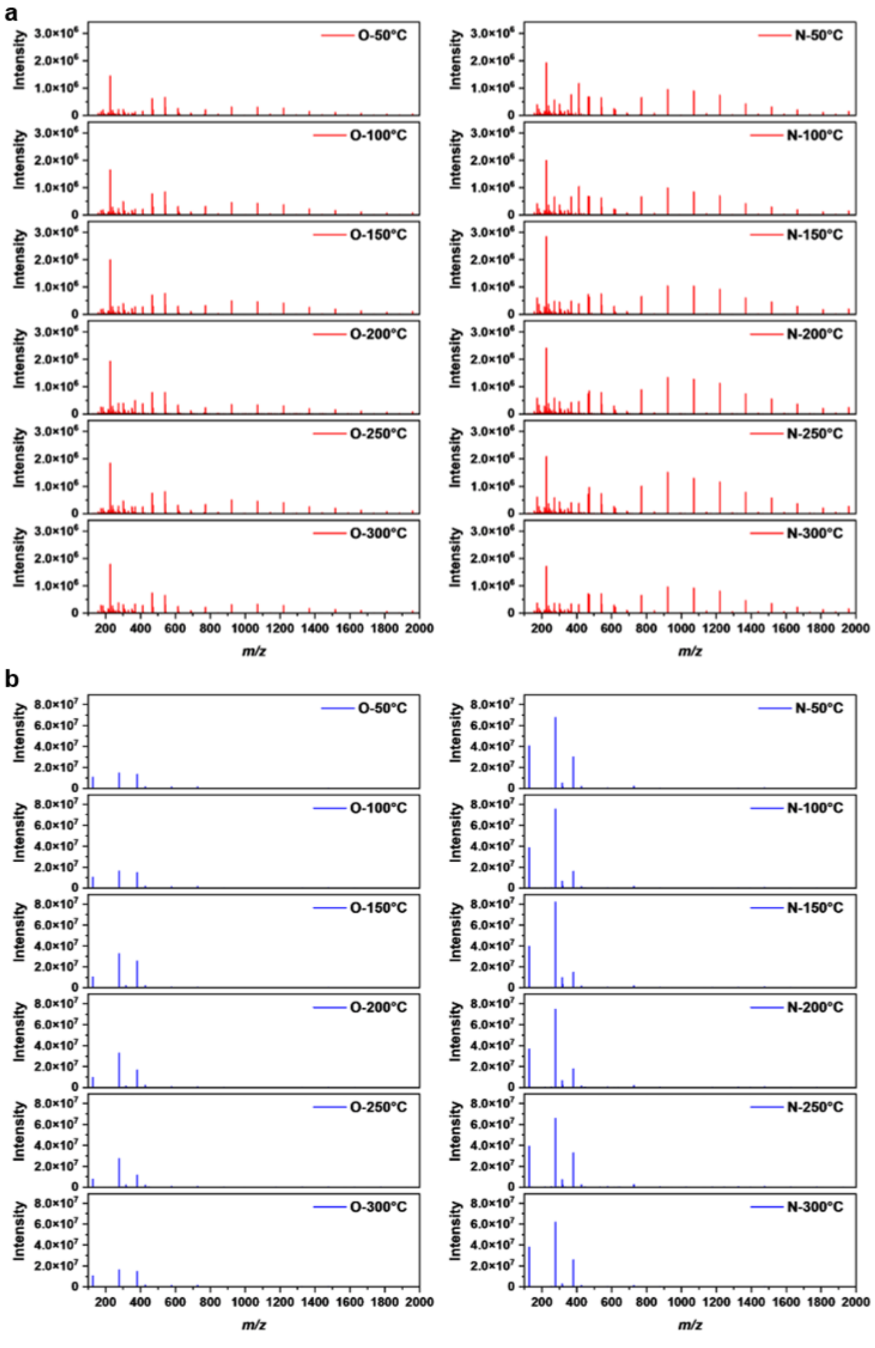

(a), (b) Mass spectra of NaI cluster ions obtained using an ion transfer tube with a length of 270 mm (O) and an ion transfer tube with a length of 120 mm (N). The spectrum in (a) was acquired in positive ion mode, and that in (b) was acquired in negative ion mode. The temperature of the ion transfer tube varied from 50 to 300 °C.

Figure S3. Comparison of the signal intensities of NaI cluster ions obtained using ion transfer tubes of different lengths.

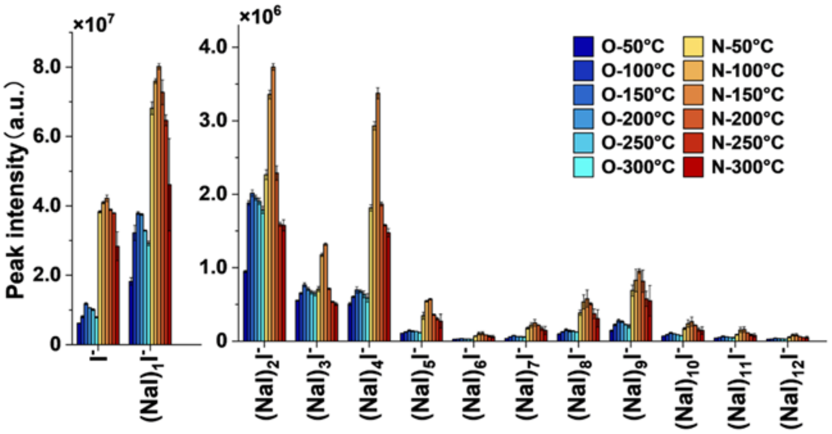

Comparison of the signal intensities of NaI cluster ions acquired in negative ion mode using ion transfer tubes of different lengths. The NaI cluster ions generated by ESI were measured using ion transfer tubes with lengths of 270 mm (O) and 120 mm (N). The temperature of the ion transfer tube was varied from 50 to 300 °C. Shortening the ion transfer tube increased the signal intensity of the NaI cluster ions by an average of 2.9-fold (1.7–4.8-fold). The ion signal intensity reached its maximum at an ion transfer tube temperature of 150 °C and increased by an average of 1.9-fold (1.5–2.6-fold) compared with that at 300 °C, which was the lowest.

Figure S4. Comparison of contact angles of diiodomethane and *n*-hexadecane on quartz substrates.

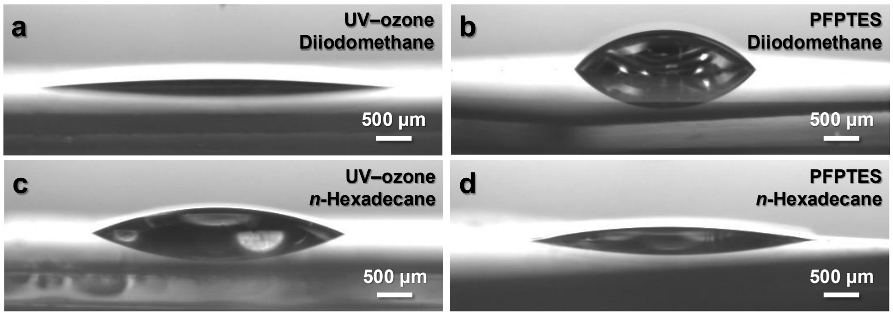

Diiodomethane and *n*-hexadecane (1 µL) were dropped onto quartz substrates treated with UV–ozone cleaning only and quartz substrates modified with PFPTES after UV–ozone cleaning, and the contact angles immediately after dropping were measured. (a), (b) Average values of five contact angle measurements for diiodomethane on the quartz substrates treated with UV–ozone cleaning and PFPTES modification were 30.9° and 48.9°, respectively. (c), (d) Average values of five contact angle measurements of *n*-hexadecane on the quartz substrates treated with UV–ozone cleaning and PFPTES modification were 20.7° and 9.5°, respectively.

Figure S5. Ion images of a mouse brain section.

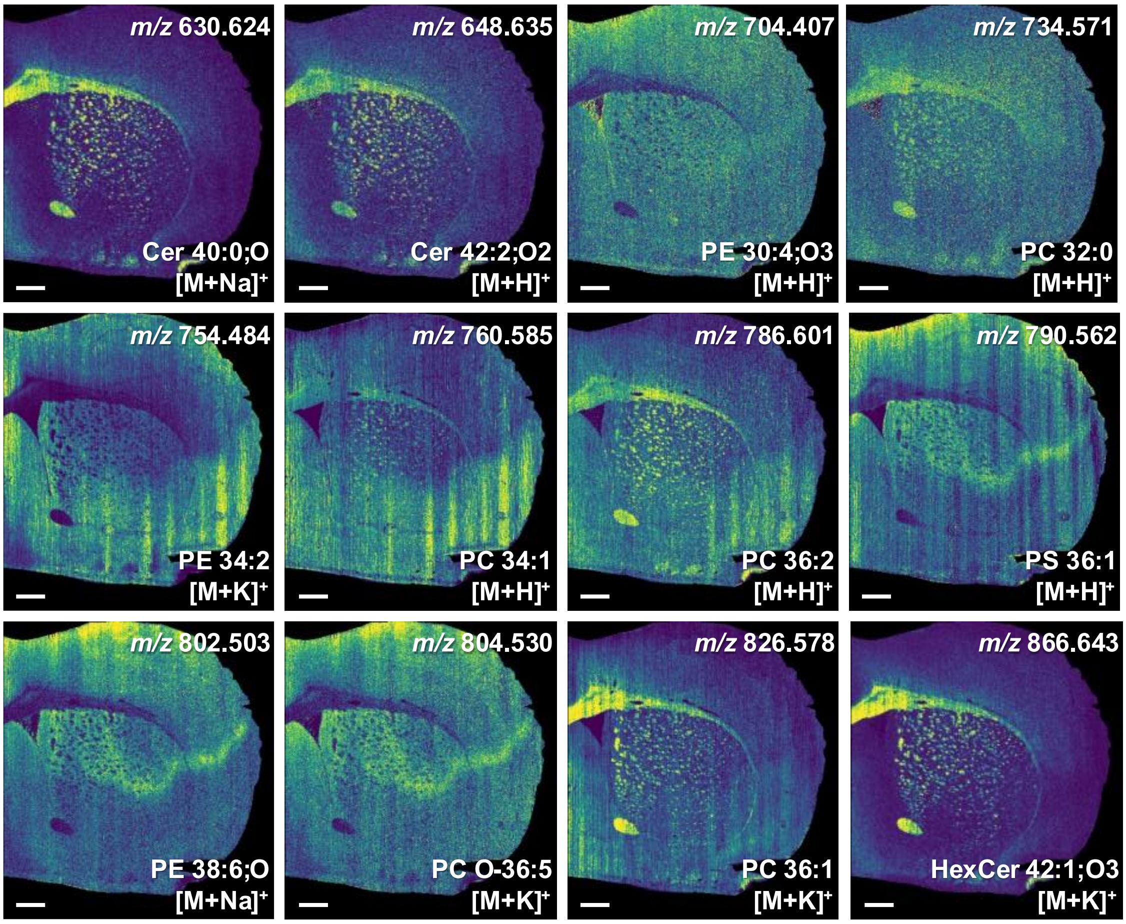

Ion images of mouse brain tissue acquired at a pixel size of 10 μm using a chemically modified probe. Scale bar: 500 μm.

Table S1. Parameters of the laser puller used to fabricate the capillary probe.

Data		HEAT	FIL	VEL	DEL	PUL
Validation of the feedback control mechanism	LINE1	230	0	10	255	0
MSI-1 (Pixel size 10 μm)	LINE1	230	0	8	255	0
MSI-2 (Pixel size 5 μm)	LINE1	220	0	8	255	0
	LINE2	210	0	7	255	0
MSI-3 (Pixel size 10 μm)	LINE1	230	0	8	255	0
MSI-4 (Pixel size 5 μm)	LINE1	220	0	10	255	0
	LINE2	220	0	8	255	0

Table S2. List of instruments used in the t-SPESI measurement system.

t-SPESI unit		
HPS80-50X-M5	Sigma Koki	Stepping motor XY stage
OSMS20-85(Z)	Sigma Koki	Stepping motor Z stage
B22-083	THK Precision	Piezo Z stage
TC20-2024A	Neoark	Laser source
Sample unit		
U-723.25	PI	Piezo XY stage
OSMS60-5ZFW	Sigma Koki	Stepping motor Z stage
Microscope unit		
CU-Mini-FL	Sigma Koki	Compact optical microscope
GIP-101B	Sigma Koki	Objective lens stage
HAX-0405	Nippon Boushin Industry	Benchtop Air Spring Isolation Table
Control and measurement unit		
ZS-6228S-8M	Zenisu Measurement System	USB-PhotoMOS Adapter
C-867.2U2	PI	Piezo XY stage controller
PH103	Nano Control	High voltage amplifier for probe oscillation
NCS7102C	THK Precision	Piezo Z stage driver
USB-6346	NI	Multifunction I/O device
HIT-M	Sigma Koki	Stepping motor Z stage driver
IC1500CU	SHODENSYA	USB camera for observation of t-SPESI unit
MLH-10X	Computar	Macro zoom lens
SHOT-702H	Sigma Koki	Stepping motor XY stage driver
PJ-1505-2CA	CCS	LED driver for microscope unit
Moku:Go	Liquid Instruments	Lock-in amplifier
Moku:Go	Liquid Instruments	PID controller
DPS-6020	Neoark	Laser controller
Precision 3460	DELL	

Table S3. Putative assignments of positive ion peaks observed in the mouse brain tissue.

m/z	Tentative assignment	Chemical formula	Ion species	Calculated *m/z*	Δ *m/z*	Error（ppm）
630.624	Cer 40:0;O	$C_{40}H_{81}NO_2P$	$[M+Na]^+$	630.6159	0.008	12.8
648.635	Cer 42:2;O2	$C_{42}H_{81}NO_3$	$[M+H]^+$	648.6289	0.006	9.4
704.407	PE 30:4;O3	$C_{35}H_{62}NO_{11}P$	$[M+H]^+$	704.4133	-0.006	8.9
734.571	PC 32:0	$C_{40}H_{80}NO_8P$	$[M+H]^+$	734.5694	0.002	2.2
754.484	PE 34:2	$C_{39}H_{74}NO_8P$	$[M+K]^+$	754.4784	0.006	7.4
760.585	PC 34:1	$C_{42}H_{82}NO_8P$	$[M+H]^+$	760.5851	0.000	0.1
786.601	PC 36:2	$C_{44}H_{82}NO_8P$	$[M+H]^+$	786.6007	0.000	0.4
790.562	PS 36:1	$C_{42}H_{80}NO_{10}P$	$[M+H]^+$	790.5593	0.003	3.4
802.503	PE 38:6;O	$C_{43}H_{74}NO_9P$	$[M+Na]^+$	802.499	0.004	5.0
804.530	PC O-36:5	$C_{44}H_{80}NO_7P$	$[M+K]^+$	804.5304	0.000	0.5
826.578	PC 36:1	$C_{44}H_{86}NO_8P$	$[M+K]^+$	826.5723	0.006	6.9
838.617	HexCer 40:1;O3	$C_{46}H_{89}NO_9$	$[M+K]^+$	838.6169	0.000	0.1
866.643	HexCer 42:1;O3	$C_{48}H_{93}NO_9$	$[M+K]^+$	866.6482	-0.005	6.0
